# Total Minimally Invasive Curative Staged Resections After Induction Systemic Therapy for Metastatic Rectal Cancer

**DOI:** 10.1002/cnr2.70051

**Published:** 2024-11-07

**Authors:** Tohru Takahashi, Takahiro Ishii, Taku Maejima, Dai Miyazaki, Susumu Fukahori, Hiroaki Kuwahara, Eriko Aimono, Taichi Kimura, Mitsuru Yanai, Masahiro Hagiwara

**Affiliations:** ^1^ Department of General Surgery Sapporo Higashi Tokushukai Hospital Sapporo Hokkaido Japan; ^2^ Department of Gastroenterology Sapporo Higashi Tokushukai Hospital Sapporo Hokkaido Japan; ^3^ Department of Respiratory Surgery Sapporo Higashi Tokushukai Hospital Sapporo Hokkaido Japan; ^4^ Department of Pathology Sapporo Higashi Tokushukai Hospital Sapporo Hokkaido Japan

**Keywords:** FOLFOXIRI plus bevacizumab, intensified triplet chemotherapy, laparoscopic staged resection, R0 resection, synchronous metastases, unresectable colorectal cancer

## Abstract

**Background:**

Intensified systemic chemotherapy following minimally invasive surgery for patients with unresectable metastatic colorectal cancer is performed to achieve curative resection and improve quality of life. We report a case of initially unresectable rectal cancer with metastases treated with laparoscopic and thoracoscopic staged resections after triplet chemotherapy plus bevacizumab.

**Case:**

A 71‐year‐old man was referred to our hospital to examine the cause of constipation. A circumferential adenocarcinoma with extramural invasion and lateral lymphadenopathy was identified in the lower rectum with simultaneous metastatic liver and lung tumors. Intensified triplet chemotherapy plus bevacizumab was conducted to treat oncologically unresectable rectal cancer to avoid positive radial margins during surgical resection. Eleven cycles of chemotherapy resulted in regression of the tumors with metastases. Laparoscopic low anterior resection with lateral lymph node dissection was performed. Three months later, laparoscopic liver resection of the posterosuperior segment was performed without complications. Finally, the patient underwent thoracoscopic‐assisted pulmonary segmentectomy of the right basal lobe. All resected tumors had negative surgical margins, and the patient has been surviving without adjuvant chemotherapy.

**Conclusion:**

Minimally invasive staged resection and intensified chemotherapy for the treatment of initially unresectable metastatic colorectal cancer should be performed by a skilled surgical team in coordination with a multidisciplinary team.

## 1 Introduction

Colorectal cancer (CRC) is the third most commonly diagnosed cancer (10%) and second most common cause of cancer‐related death (9.4%), with an estimated 935 000 worldwide deaths in 2020 [1]. Synchronous metastases are observed in approximately 18% of patients with CRC at initial diagnosis [2].

Systemic chemotherapy is widely approved as the standard treatment for initially unresectable metastatic colorectal cancer (mCRC). Although doublet chemotherapy plus bevacizumab is recommended as the first‐line treatment in patients with mCRC, recent studies have shown that intensified triplet chemotherapy plus bevacizumab improves progression‐free survival and objective response rate, resulting in a higher resection rate compared with doublet chemotherapy [3, 4]. To this end, this triplet regimen is offered to selected patients as an optional first‐line therapy following shared decision‐making [5].

Laparoscopic surgery for CRC is beneficial because it results in less morbidity and faster recovery than open surgery, while yielding equivalent oncological outcomes [6]. Laparoscopic procedures have been applied to liver resection with technical advances, such as improved visibility, bleeding management, and tissue dissection [7], leading to the development of total laparoscopic strategies for managing CRC with synchronous liver metastasis (SLM) [8]. However, the optimal strategy for staged or simultaneous liver resection remains controversial in terms of patient selection and long‐term oncological outcomes [9].

We report a case of curative staged resections, using laparoscopic and thoracoscopic approaches for initially unresectable rectal cancer with liver and lung metastases, who was preoperatively treated with intensified triplet chemotherapy plus bevacizumab.

## 2 Case

**FIGURE 1:**
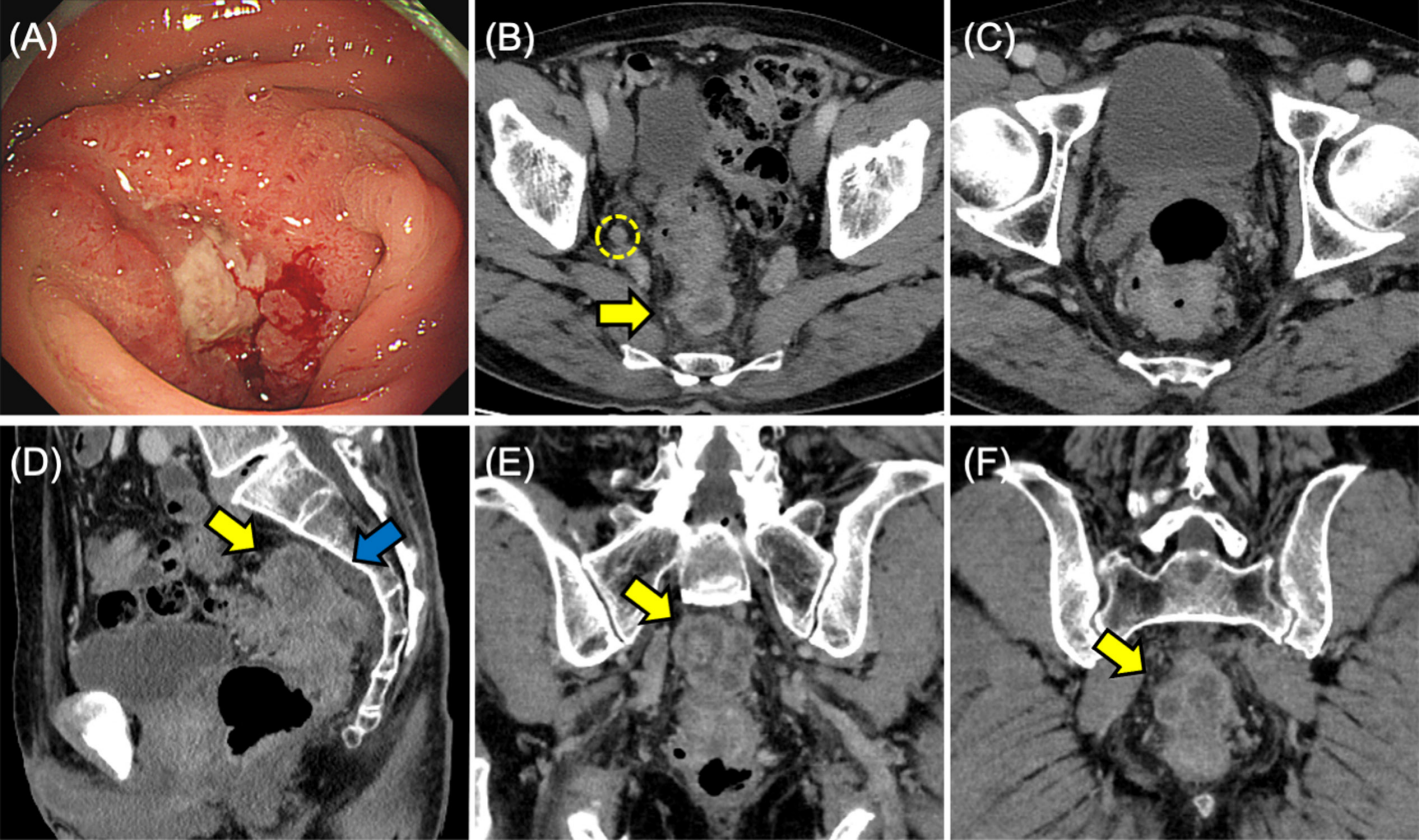
Initial diagnostic images of the rectal tumor before treatment. (A) Colonoscopy showing a circumferential tumor of the lower rectum. (B–F) Computed tomography demonstrating a rectal tumor with extramural infiltration (yellow arrows) accompanied by fluid collection (blue arrow) and lateral lymph node adenopathy (yellow dotted circle).

**FIGURE 2:**
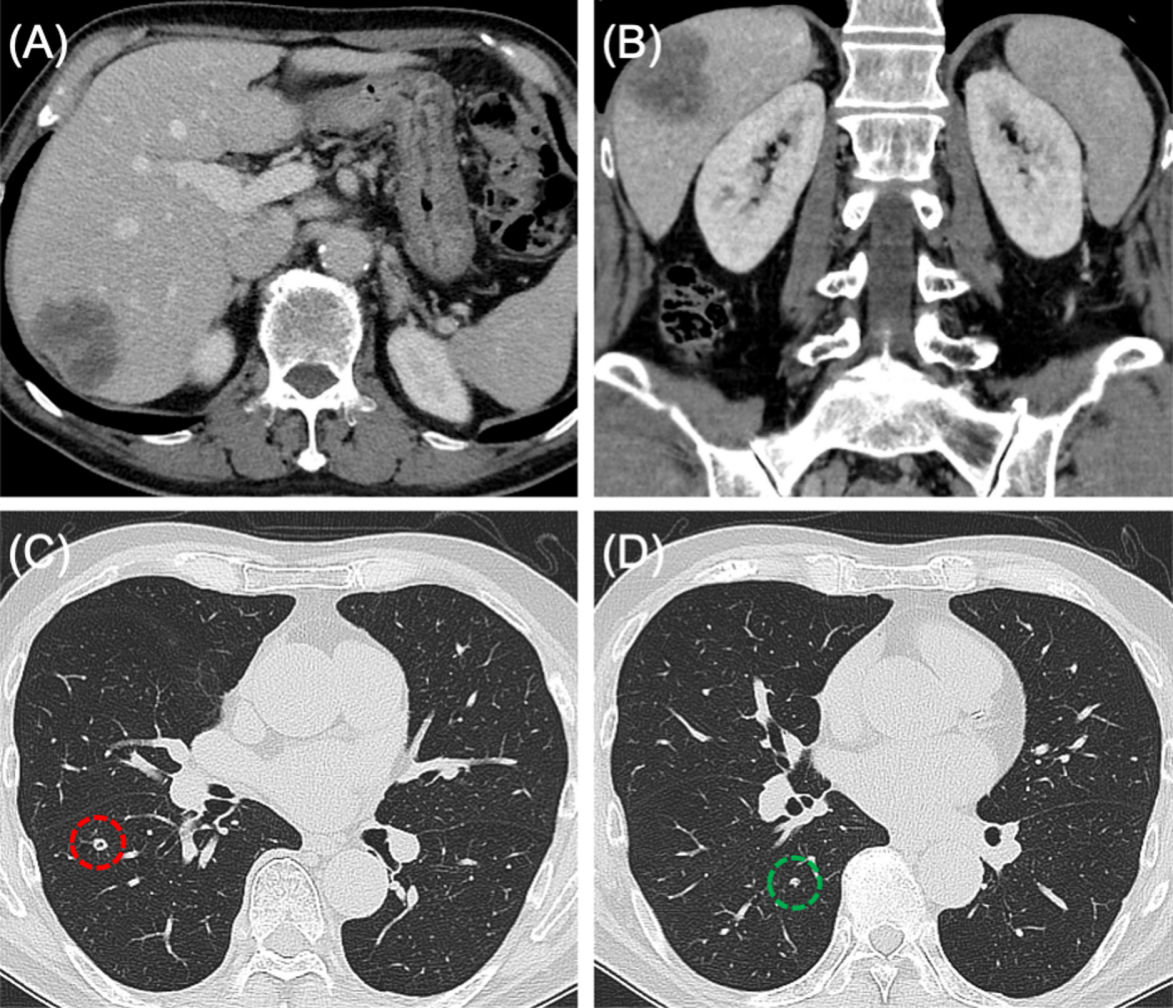
Initial diagnostic images of the metastatic tumors before treatment. Computed tomography demonstrating a solitary metastatic liver tumor (4.3 × 3.3 cm) in the posterosuperior segment (A, B) and two lung tumors in the right basal lobe (red and green dotted circles) (C, D).

**FIGURE 3:**
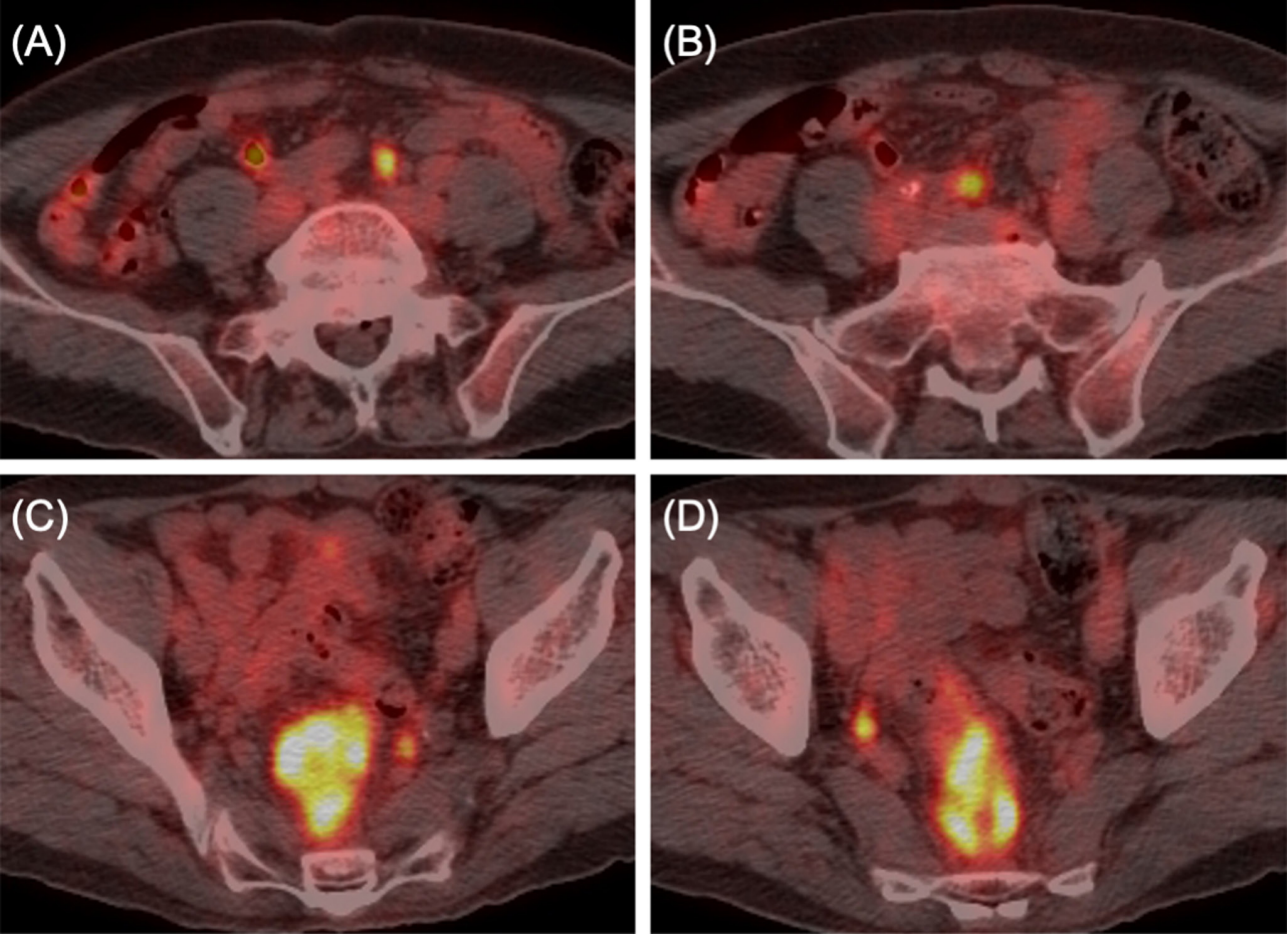
Positron emission tomography‐computed tomography images. Uptake of ^18^F‐fluorodeoxyglucose in multiple lateral lymph nodes identified in the bilateral common iliac regions (A, B) and bilateral internal iliac regions with accumulation in the tumor (C, D) before chemotherapy.

A 71‐year‐old man with a history of hypertension was referred to Sapporo Higashi Tokushukai Hospital because of constipation in May 2022. Colonoscopy identified a circumferential tumor in the rectum that could not be passed through. Biopsy results indicated moderately differentiated tubular adenocarcinoma with KRAS mutation, wild‐type BRAF, and microsatellite stability (Figure 1A). Enhanced computed tomography (CT) revealed longitudinal extended circumferential rectal wall thickening with extramural tumor infiltration, adjacent to the sacrum, with fluid collection and accompanied by scuffing into the surrounding tissues, indicating tumor invasion (Figure 1B–F). CT also revealed one metastatic liver tumor in the posterosuperior segment and two metastatic pulmonary tumors with cavernous formation in the right basal segment (Figure 2). Several swollen lateral lymph nodes (LLNs) were detected by positron emission tomography‐CT (Figure 3). Surgical resection of the rectal tumor was considered to pose a high risk of positive radial margins. Therefore, the patient was diagnosed with oncologically unresectable mCRC with microsatellite stability, performance status 0 according to the Eastern Cooperative Oncology Group, and normal body mass index (BMI, 20.62 kg/m^2^) under 75 years of age. Our multidisciplinary team recommended intensified triplet first‐line chemotherapy for initially unresectable CRC with synchronous distant metastases to expect a higher resection rate in accordance with the American Society of Clinical Oncology guidelines [5].

**FIGURE 4:**
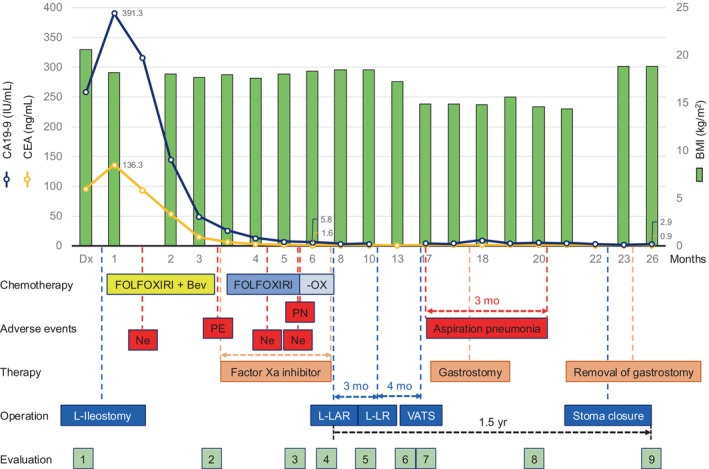
Time course of chemotherapeutic and surgical interventions with relevant adverse events, the transition of tumor marker values, and body mass index. CEA, carcinoembryonic antigen; CA19‐9, carbohydrate antigen 19–9; BMI, body mass index; Dx, initial diagnosis; FOLFOXIRI, 5‐fluorouracil, leucovorin, oxaliplatin, and irinotecan; Bev, bevacizumab; –OX, FOLFOXIRI without oxaliplatin; Ne, Neutropenia; PE, pulmonary embolism; PN, peripheral neuropathy; mo, months; L‐Ileostomy, laparoscopic ileostomy; L‐LAR, laparoscopic low anterior resection; L‐LR, laparoscopic liver resection; VATS, video‐assisted thoracic surgery. Evaluation 1: Initial diagnosis. Evaluation 2–3: CT after four cycles of FOLFOXIRI+bevacizumab (2) or four cycles of FOLFOXIRI alone (3). Evaluation 4–6: CT before surgery, L‐LAR (4), L‐LR (5), and VATS (6). Evaluation 7: CT for identifying pneumonia. Evaluation 8–9: CT for follow‐up 1 year and 1.5 years after the rectal resection.

**FIGURE 5:**
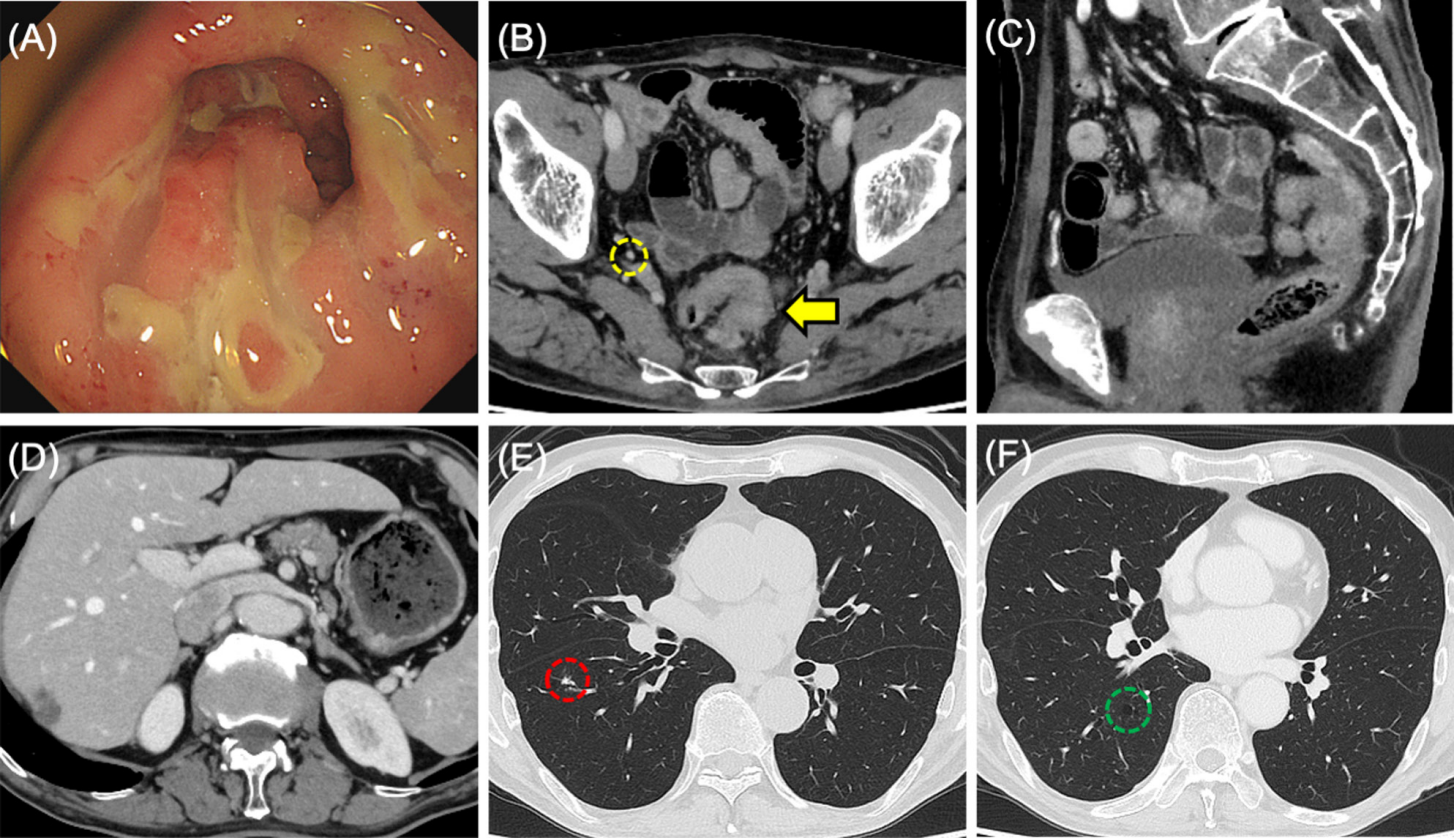
Preoperative diagnostic images after chemotherapy. (A) Colonoscopy showing findings that suggest tumor shrinkage with narrow patency of the rectal lumen. (B–F) Computed tomography showing a shrunk lateral lymph node in the right internal iliac region (yellow dotted circle) with a reduction of the primary rectal tumor (yellow arrow) (B, C). Additionally, the size of the metastatic liver tumor decreases to 1.7 × 1.2 cm (D), and the metastatic lung tumor becomes a radial scar (red dotted circle) and vacuolization (green dotted circle) (E and F, respectively).

After laparoscopic ileostomy, the patient received triplet chemotherapy [FOLFOXIRI: oxaliplatin (85 mg/m^2^, a 120‐min infusion), leucovorin (200 mg/m^2^, a 120‐min infusion), irinotecan (125 mg/m^2^, a 60‐min infusion), and 5‐fluorouracil (3200 mg/m^2^, a 48‐h continuous infusion)] plus bevacizumab (5 mg/kg, a 30‐min infusion) every 2 weeks. The patient required dose reduction due to grade 4 neutropenia after one cycle of FOLFOXIRI with bevacizumab. After four cycles of FOLFOXIRI with bevacizumab, CT showed a partial tumor response with the development of right pulmonary thromboembolism (Figure S1); therefore, the patient received thrombolytic therapy with a factor Xa inhibitor with chemotherapy discontinuation for 1 week. Chemotherapy was then resumed with FOLFOXIRI without bevacizumab and accompanied by thrombolytic therapy, resulting in the complete disappearance of the thromboembolism after four cycles of FOLFOXIRI alone (Figure S2). Subsequently, the patient developed grade 1 peripheral neuropathy. Therefore, another three cycles of chemotherapy were administered without oxaliplatin until surgery at his request (Figure 4). The tumor marker levels decreased during chemotherapy, especially when FOLFOXIRI with bevacizumab was administered (carcinoembryonic antigen decreased from 136.3 to 1.6 ng/mL; carbohydrate antigen 19–9 decreased from 391.3 to 5.8 IU/mL; Figure 4). After 11 cycles of chemotherapy, colonoscopy and CT indicated remarkable tumor shrinkage in the rectum (Figure 5A,B), and some LLNs disappeared, except for a much reduced lymph node in the right internal iliac region (Figure 5B). Rectal tumor resection was considered as the extramural tumor invasion had decreased, resulting in some distance from the sacrum (Figure 5C). Metastatic tumors in the liver and lung were also reduced in size on CT (Figure 5D–F), indicating partial tumor response. Tumor reduction was remarkably achieved following FOLFOXIRI with bevacizumab (Figure S1). Similarly, chemotherapy alone also resulted in effective tumor reduction until surgery, even if bevacizumab or oxaliplatin was discontinued (Figure S2 and Figure 5). BMI was maintained at around 18 kg/m^2^ throughout chemotherapy administration (Figure 4).

**FIGURE 6:**
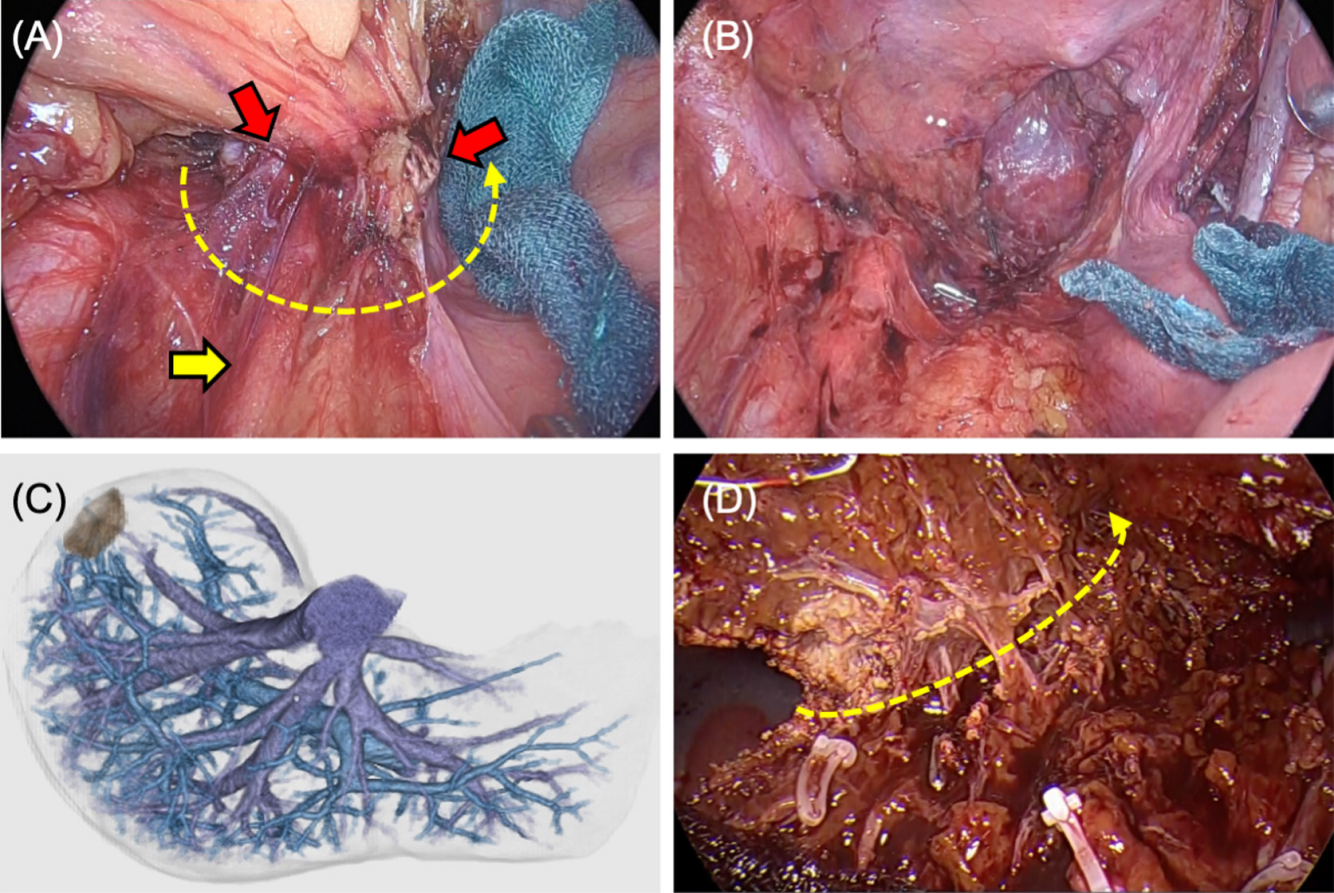
Intraoperative views and preoperative computed tomography image of the metastatic liver tumor. (A) Extramural tumor infiltration (red arrow) is identified with hypogastric nerve invasion (yellow arrow), leading to combined resection for a secure surgical margin. (B) Laparoscopic low anterior resection showing total mesorectal excision along with right lateral lymph node dissection. (C) Three‐dimensional computed tomography image showing the site of the metastatic liver tumor in the posterosuperior segment. (D) Laparoscopic partial liver resection in the posterosuperior segment is performed using intracorporeal ultrasonography with secured resection margins. The yellow dotted line with an arrow represents the resection line (A, D).

After 5 months of chemotherapy administration, the patient underwent laparoscopic low anterior resection with right LLN dissection (Figure 6A,B). The patient recovered without complications and was discharged on postoperative day 10. After 3 months of physical recovery, the patient underwent laparoscopic partial liver resection and was discharged on postoperative day 5 without complications (Figure 6C,D). Minimally invasive conversion surgery comprising thoracoscopic‐assisted pulmonary segmentectomy was performed 4 months after liver resection; the patient was discharged on postoperative day 10 without complications.

**FIGURE 7:**
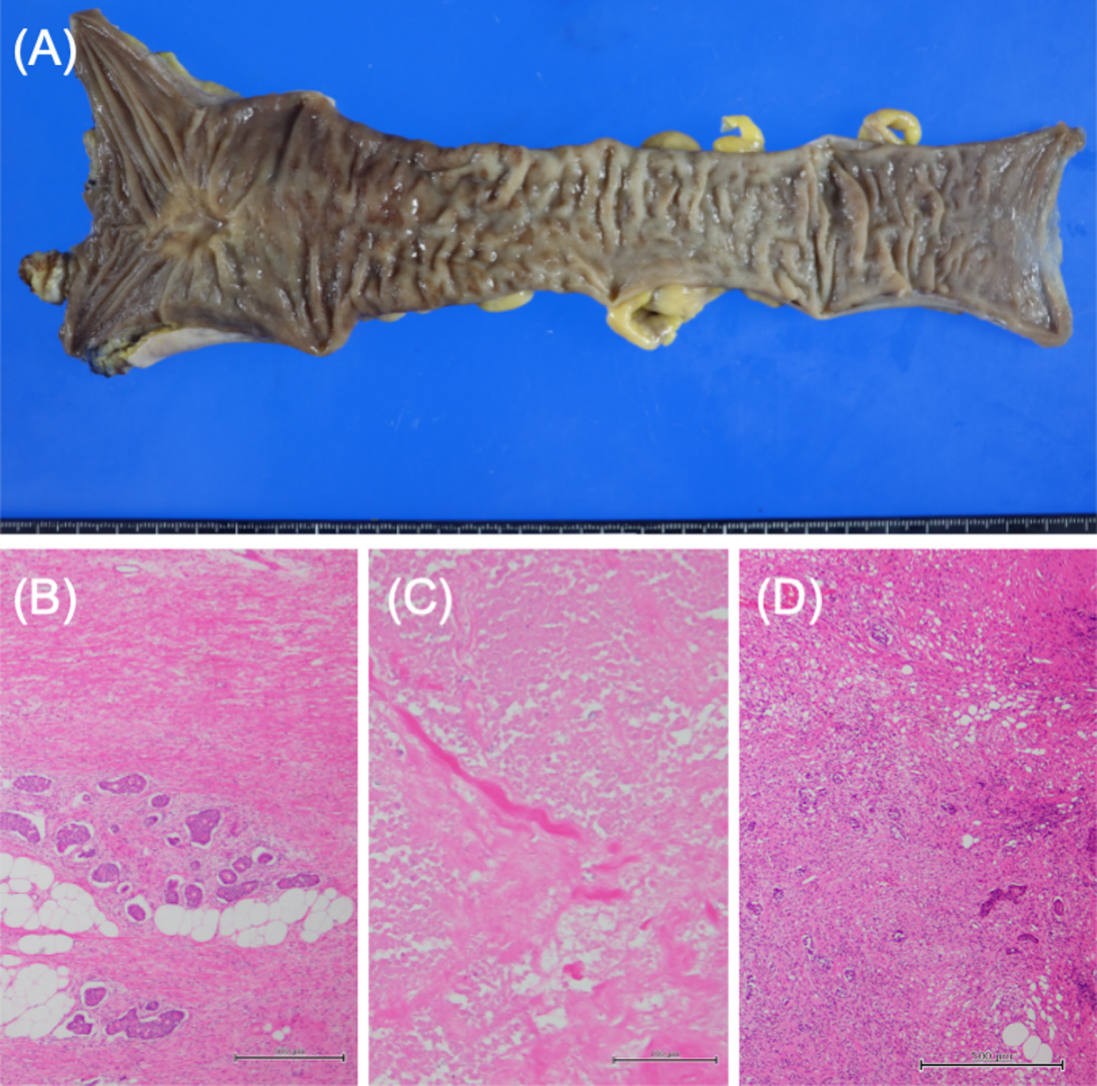
Macroscopic image and histopathological examinations of the resected rectum and lateral lymph node. (A) The resected rectum reveals a tumor on the serosal surface of the posterior wall. (B–D) Histopathological findings reveal that more than two‐thirds of the tumor area is replaced by fibrosis accompanied by necrosis in the rectum (B, ×40; C, ×200) and in one lateral lymph node (D, ×40). The scale bars represent 500 μm (B, D) and 100 μm (C).

**FIGURE 8:**
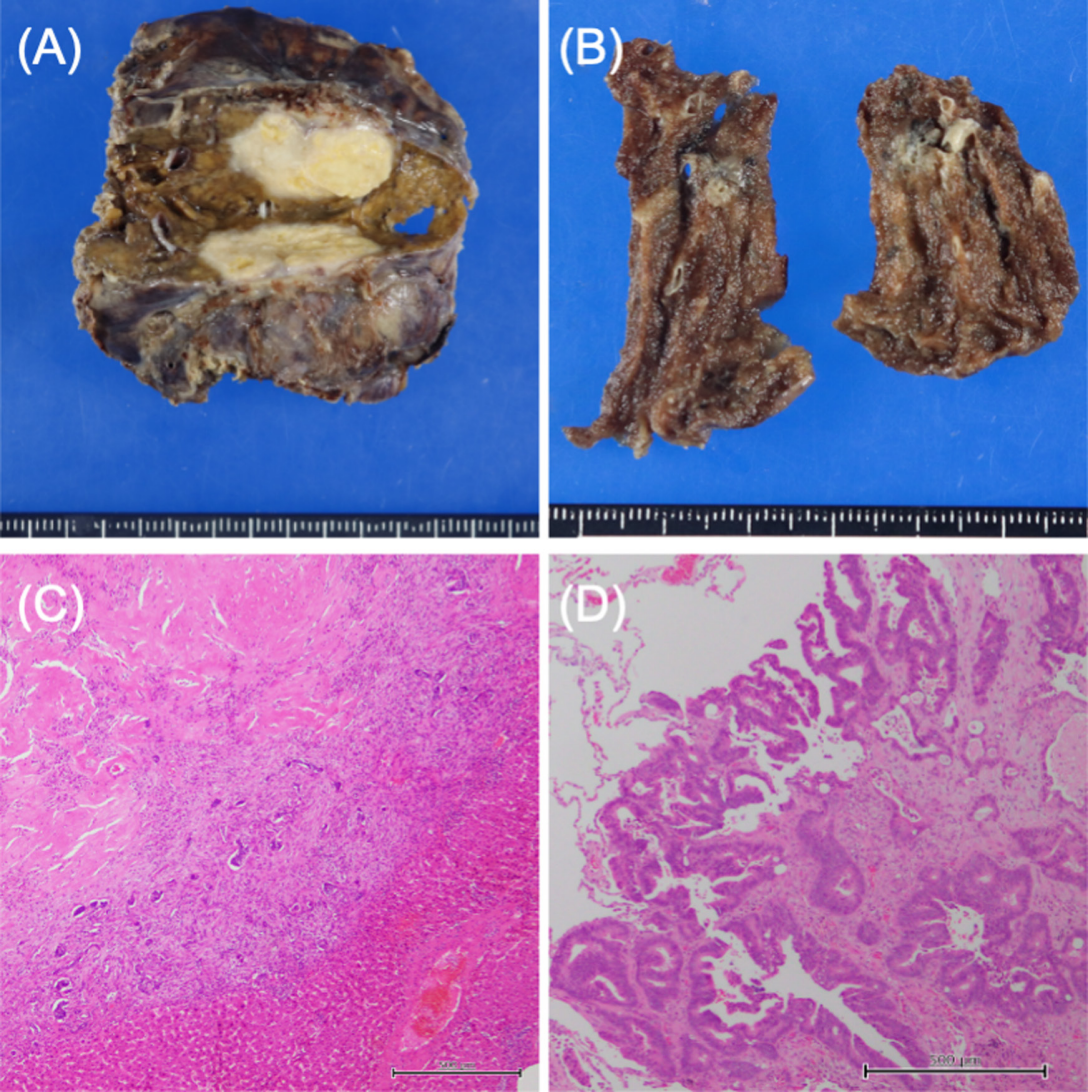
Macroscopic images and histopathological examinations of the resected specimens for the metastatic tumors. (A, B) Surgical margins of the resected tumors are identified as negative in the liver (A) and lung (B). (C, D) Histopathological findings reveal massive coagulative necrosis accompanied by fibrosis with less than 30% of viable tumor cells in the liver (C, ×40). However, over 60% of remnant tumor cells remain viable in two pulmonary metastases (D, ×40). The scale bar represents 500 μm.

Macroscopic examination of the resected rectum revealed an exposed tumor on the serosal surface of the posterior wall (Figure 7A). The microscopic findings showed that more than two‐thirds of the tumor area had been replaced by fibrosis accompanied by necrosis, although viable tumor cells remained in the rectum and the LLNs (Figure 7B–D). Regarding the metastatic tumors, surgical‐free margins of the tumors were identified macroscopically in the liver and lung (Figure 8A,B, respectively). Microscopic examination revealed massive coagulative necrosis accompanying fibrosis, with less than 30% of viable tumor cells remaining in the liver (Figure 8C). However, over 60% of remnant tumor cells remained viable in the two pulmonary metastases (Figure 8D).

The patient developed refractory aspiration pneumonia 2 months after pulmonary resection and underwent gastrostomy to prevent aspiration and maintain nutritional status (BMI, 14.4–15.6 kg/m^2^). After 3 months, he recovered from pneumonia; then, he finally underwent closure of intestinal stoma and gastrostomy, leading to nutritional and body weight improvement (BMI, 18.8 kg/m^2^). Although the patient did not receive adjuvant chemotherapy during treatment, he has been surviving without stoma management. Outpatient clinic follow‐up revealed normal tumor markers and CT scan findings indicating no evidence of recurrence (carcinoembryonic antigen, 0.9 ng/mL; carbohydrate antigen 19–9, 2.9 IU/mL) for more than 1.5 years since the primary rectal tumor was resected (Figure 4).

## 3 Discussion

Triplet chemotherapy (FOLFOXIRI) plus bevacizumab is a promising regimen in patients with initially unresectable mCRC. Two randomized controlled trials compared FOLFOXIRI plus bevacizumab with doublet chemotherapy plus bevacizumab, resulting in improved progression‐free survival (FOLFOXIRI: 19.2 and 10.6 months vs. doublet therapy: 16.4 and 9 months) and a higher response rate (FOLFOXIRI: 62% and 54% vs. doublet therapy: 50% and 33%), leading to better microscopic complete resection (R0) rates (FOLFOXIRI: 17% and 51% vs. doublet therapy: 12% and 37%) [3, 4]. A higher response rate can predict the resectability of initially unresectable mCRC confined to the liver [10]. One systematic review and pooled analysis of FOLFOXIRI plus bevacizumab reported a pooled objective response rate of 69%, with higher overall and R0 resection rates in patients with liver‐limited metastases (62.2% and 54.7%, respectively) than in those with multiple‐site metastases (28.7% and 16.9%, respectively) [11]. Therefore, intensive and effective chemotherapy increases the opportunity of conversion surgery for initially unresectable mCRC and improves progression‐free and overall survival.

The timing of surgical resection should be considered when intensified chemotherapy is effective in managing unresectable mCRC. Conventionally, a staged strategy comprising initial resection of the primary tumor followed by liver resection has been performed for CRC with SLM. A recent study has reported that simultaneous resection using a total laparoscopic procedure resulted in a shorter operating time compared with laparoscopic staged resection for treating CRC with SLM, with similar morbidity rates [8]. In contrast, laparoscopic staged resections, with initial resection of CRC, have shown acceptable midterm oncological outcomes and low severe morbidity in patients with CRC and liver metastasis [9]. Moreover, the only relevant prospective, randomized, controlled trial reported the cautious application of simultaneous resection for major hepatectomy and complicated colorectal resections; however, the major complication rate of simultaneous resection was equivalent to that of staged resection among patients with resectable CRC and SLM [12]. The patient had initially unresectable rectal cancer with LLN metastasis requiring complicated primary resection after systemic chemotherapy. Moreover, a metastatic liver tumor located in the posterosuperior segment demands highly advanced techniques and a longer time to perform laparoscopic resection. In addition, a low BMI was considered indicative of poor tolerability for extensive surgery. Therefore, laparoscopic simultaneous resection was cautiously applied; staged resection was the only viable option.

The limitations of this study include the relatively short follow‐up period of 1.5 years after primary resection or less than 1 year after pulmonary resection. Furthermore, another limitation is that resectability depends on chemotherapy response rate, patient compliance, and physical tolerance to several operative procedures. Therefore, laparoscopic and thoracoscopic staged resections after intensified triplet chemotherapy are optimal in patients with mCRC because they reduce physical waste and allow curative resection; nonetheless, they necessitate careful observation of disease progression during treatment.

In conclusion, minimally invasive surgery comprising staged resection using total laparoscopic and thoracoscopic techniques alleviated the invasiveness of simultaneous resection of CRC with SLM and allowed R0 resection after FOFOXIRI plus bevacizumab treatment. Although careful surveillance is necessary, a strategy involving precise staged resection using a minimally invasive technique and intensified chemotherapy for the treatment of initially unresectable mCRC could improve survival. Therefore, this strategy should be considered by a highly skilled and experienced laparoscopic surgical team in coordination with a multidisciplinary team.

## Author Contributions

**Tohru Takahashi:** conceptualization, methodology, investigation, data curation, resources, writing – original draft, writing – review and editing, project administration, supervision. **Takahiro Ishii:** conceptualization, methodology, investigation, resources. **Taku Maejima:** investigation, resources. **Dai Miyazaki:** conceptualization, data curation, visualization. **Susumu Fukahori:** conceptualization, data curation. **Hiroaki Kuwahara:** methodology, data curation. **Eriko Aimono:** conceptualization, data curation, writing – review and editing. **Taichi Kimura:** conceptualization, investigation, data curation. **Mitsuru Yanai:** conceptualization, resources, visualization. **Masahiro Hagiwara:** conceptualization, methodology, project administration.

## Ethics Statement

All procedures followed in this study were performed in accordance with the ethical standards laid down in the 1964 Declaration of Helsinki and its subsequent amendments.

## Consent

Informed consent was obtained from the patient for the publication of this study.

## Conflicts of Interest

The authors declare no conflicts of interest.

## Supporting information


**Figure S1.** Computed tomography images after four cycles of FOLFOXIRI with bevacizumab. They reveal shrinkage of the rectal tumor with extensive reduced extramural infiltration (yellow arrow) (A, B). The metastatic liver tumor also decreases to 3.0 × 2.6 cm (C) with a reduction in the metastatic lung tumors (red and green dotted circles) (D, E), indicating partial tumor response in the rectum and liver. Computed tomography image revealing pulmonary arterial thrombosis (red arrow) in the right inferior branches simultaneously (F).

**Figure S2.** Computed tomography images after four cycles of FOLFOXIRI alone. Compared with the images after four cycles of FOLFOXIRI with bevacizumab (Figure S1), extramural infiltration of the rectal tumor (yellow arrow) is further reduced (A, B), with a further reduced size (2.6 × 1.9 cm) in the metastatic liver tumor (C), but with similar sizes in the two lung tumors (red and green dotted circles) (D, E), indicating stable disease in all tumor sites. The pulmonary arterial thrombosis (red arrow) completely disappeared following thrombolytic therapy (F).

## Data Availability

The data that support the findings of this study are available from the corresponding author upon reasonable request.
