# Supplementary figures and images for: Total Minimally Invasive Curative Staged Resections After Induction Systemic Therapy for Metastatic Rectal Cancer
Source: Cancer Rep (Hoboken). 2024 Nov 7;7(11):e70051. doi: 10.1002/cnr2.70051 (PMC11541059; doi:10.1002/cnr2.70051)

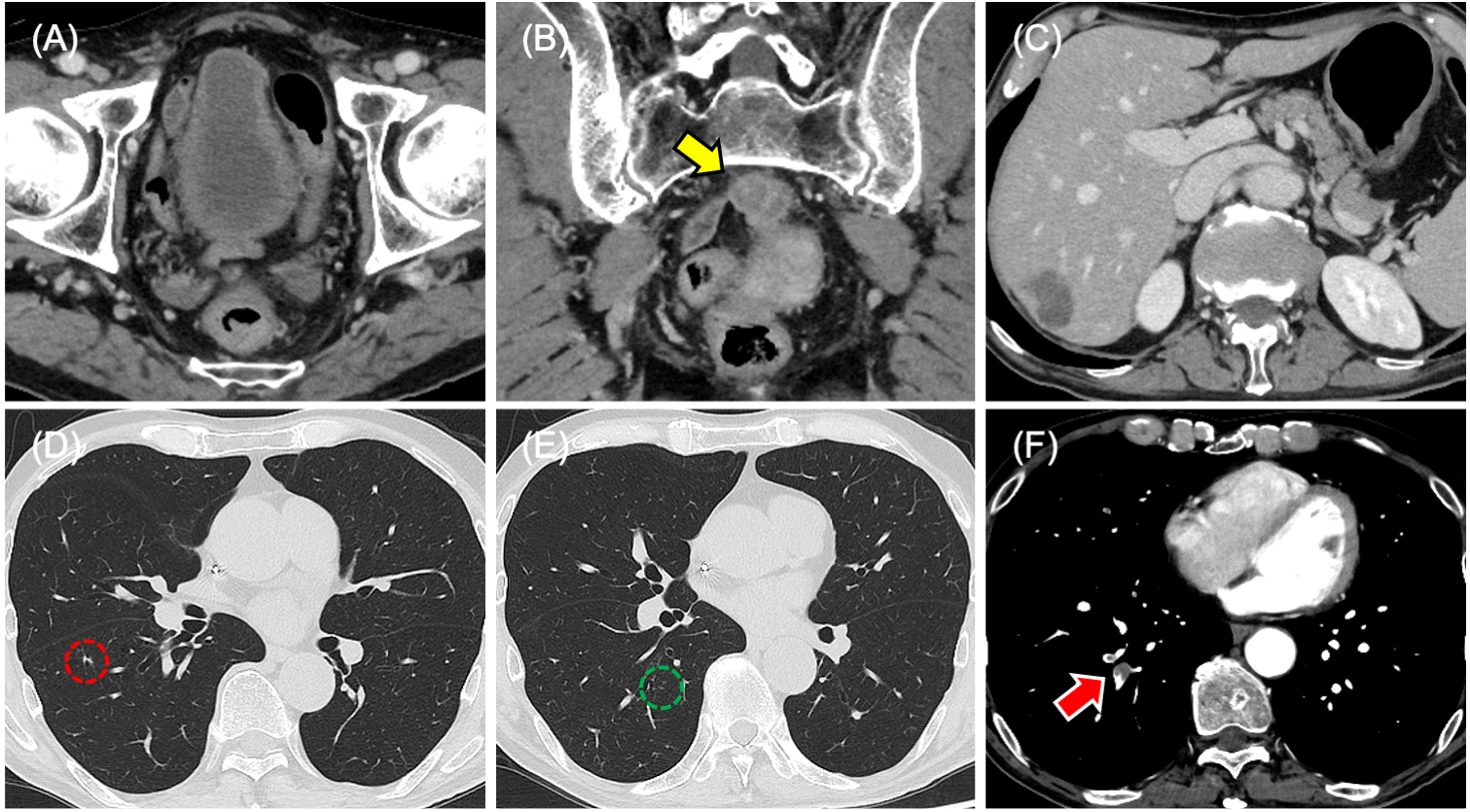

Supplement: Supplementary file 1 — Figure S1. Computed tomography images after four cycles of FOLFOXIRI with bevacizumab. They reveal shrinkage of the rectal tumor with extensive reduced extramural infiltration (yellow arrow) (A, B). The metastatic liver tumor also decreases to 3.0 × 2.6 cm (C) with a reduction in the metastatic lung tumors (red and green dotted circles) (D, E), indicating partial tumor response in the rectum and liver. Computed tomography image revealing pulmonary arterial thrombosis (red arrow) in the right inferior branches simultaneously (F). [file CNR2-7-e70051-s002.tiff]

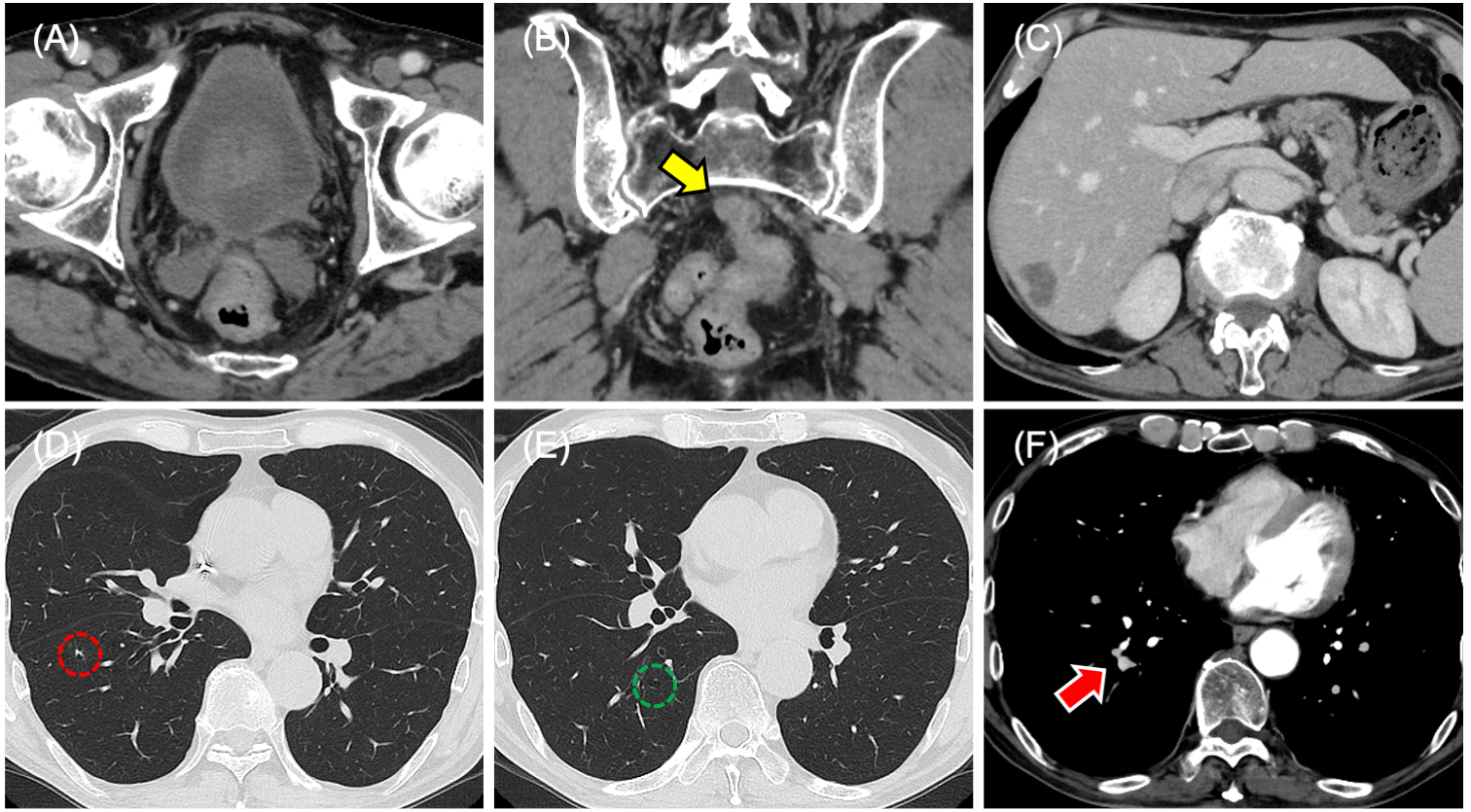

Supplement: Supplementary file 2 — Figure S2. Computed tomography images after four cycles of FOLFOXIRI alone. Compared with the images after four cycles of FOLFOXIRI with bevacizumab (Figure S1), extramural infiltration of the rectal tumor (yellow arrow) is further reduced (A, B), with a further reduced size (2.6 × 1.9 cm) in the metastatic liver tumor (C), but with similar sizes in the two lung tumors (red and green dotted circles) (D, E), indicating stable disease in all tumor sites. The pulmonary arterial thrombosis (red arrow) completely disappeared following thrombolytic therapy (F). [file CNR2-7-e70051-s001.tiff]
